# Exercise training has restorative potential on myocardial energy metabolism in rats with chronic heart failure

**DOI:** 10.22038/IJBMS.2018.29294.7076

**Published:** 2018-08

**Authors:** Li Wang, Kai Gao, Dan Wang

**Affiliations:** 1 Department of Physical Education, Shandong University, Weihai, Weihai 264209, Shandong Province, China; 2 Medical Imaging Faculty, Weifang Medical University, Weifang 261053, Shandong Province, China; 3 Division of physical education, General course Teaching Department, Weifang Medical University, Weifang 261053, Shandong Province, China

**Keywords:** Basal metabolism, Blood pressure, Exercise, Fatty acid, Heart failure, Myocardial infarction

## Abstract

**Objective(s):**

Exercise training is a well-known accelerator for the treatment of chronic heart failure (CHF). The current study aimed to investigate the restorative effects of aerobic interval training (AIT) intervention on myocardial energy metabolism in CHF rats.

**Materials and Methods:**

Post-myocardial infarction (MI) heart failure animal model was established. The Sprague-Dawley rats were randomly divided into sham operation group (Sham group), CHF model group, and CHF exercise group (Exercise-CHF group).

**Results:**

Our data showed that when compared to the Sham group, the left ventricular systolic pressure (LVSP), myocardial glycogen content, and expression levels of key components of AMP-activated protein kinase (AMPK) pathway were decreased significantly (*P<*0.05) in the CHF-model group, while the left ventricular end diastolic pressure (LVEDP), fatty acid (FA) concentration, lactic acid content, and AMPKα phosphorylation (p-AMPKα) were increased significantly (*P<*0.05) in the CHF-model group. Importantly, AIT reversed these alterations induced by post-MI.

**Conclusion:**

Findings of this study demonstrated that AIT could improve the metabolic remodeling and enhance cardiac function, which may be associated with the activation of AMPK/ peroxisome proliferator activated receptor α (PPARα) and its downstream signaling pathway.

## Introduction

Heart failure (HF) is a common endpoint of many forms of cardiovascular disease and a leading cause of morbidity and mortality in older individuals (1). Chronic heart failure (CHF) is a complex syndrome characterized by the inability of cardiac muscle to maintain peripheral tissue blood supply, therefore leading to impairment of the whole body energetic metabolism. Metabolic remodeling of myocardial cells is critical for the development of CHF. It has been reported that myocardium metabolic remodeling accelerates the CHF process (2). Accumulated evidences demonstrate that aerobic interval training (AIT) is an effective strategy for prevention and treatment of cardiovascular diseases (3), being an efficient adjuvant therapy for CHF. AIT improves exercise tolerance and cardiac contractility, which are associated with alterations of cardiac Ca^2+^ handling (4). AIT has been showed to improve myocardial metabolism in patients with CHF; however, the underlying molecular mechanism remains unclear. In addition, the cellular basis of associative therapy on myocardial energy metabolism has not been clarified yet. It has been reported that AMP-activated protein kinase (AMPK) plays important roles in the development of the heart and myocardial metabolism, as well as in myocardial ischemia reperfusion injury (5). Current researches on the relationship between exercise and AMPK are mainly studied in skeletal muscle tissue, but less in myocardium. In this study, using a chronic myocardial infarction (MI) CHF rat model, the effects of AIT exercise on myocardial energy metabolism was evaluated, and the underlying mechanism was also explored.

## Materials and Methods

***Establishment of chronic heart failure model following myocardial infarction***

A total of 60 male Sprague-Dawley (SD) rats (8-10 weeks of age with weights of 260~320 g) maintained under specific pathogen-free (SPF) condition, were provided by the experimental animal center of Shanghai (Shanghai, China). Rats were housed at a temperature of 22–25 ^°^C in a 50–70% humidity controlled room with a 12 hr light/dark cycle. A standard rat diet including tap water was given *ad libitum* throughout the experimental period. Rats were numbered in the ascending order and randomly assigned to three groups, the control group (Sham group) and two experimental groups (CHF-Model group and Exercise-CHF group). The Sham group (n=20) and CHF-Model group (n=20) were kept in a quiet state, and the Exercise-CHF group (n=20) was trained for 8 weeks. CHF model was established by coronary artery ligation method as described previously (6). The respirator is connected to a vaporizer to supply 0.5 to 1.0% Isoflurane (Baxter) mixed with 100% oxygen to maintain general anesthesia. Thoracotomy was performed to expose the heart after anesthesia. Left anterior descending coronary artery was ligated by 0 silk thread 2~3 mm below the left atrial appendage. In the CHF group, after sodium pentobarbital anesthesia, multi-channel physiological signal acquisition and processing system was used to record the limb lead electrocardiogram (ECG). After MI induced by ligation of the left anterior descending coronary artery, S-T segment elevation or T wave inversion phenomenon was observed by ECG, which was considered as successful modeling. The CHF model was induced for 4 weeks feeding in the cage. After model establishment, rats were randomly divided into two groups, the CHF-Model group and the Exercise-CHF group. In the Sham group, the rats were treated only with chest opening and threading but without ligation, and the other treatments were the same as those in the CHF group. All animal studies were approved by the Weifang Medical University Institutional Animal Care and Use Committee (approval number: WMU016-24).

***Aerobic interval training protocol***

Our training model was slightly modified based on a previous report (7). Rats in the Exercise-CHF group underwent treadmill exercise (on a flat) 4 weeks after operation. In formal training, the running sessions started with a 10 min warm up at 40% VO_2_max, and consisted of successive 4 min exercise periods at 85-90% of VO_2_max, followed by 6 min recovery periods at 50-65% of VO_2_max. The intensities and length of the training were gradually incremented every week. The total exercise time was 60 min. The training was performed for 5 days a week and a total of 8 weeks.

***Measurement of hemodynamic parameters***

Rats were anesthetized via intraperitoneal injection with sodium pentobarbital. Cardiac catheter was inserted into the left ventricle, and a pressure transducer was connected. The multimedia biological signal acquisition and processing system was used to record mean arterial pressure (MAP), the left ventricular systolic pressure (LVSP), the left ventricular end-diastolic pressure (LVEDP), left ventricular peak velocities of contraction (positive dP/dt_max_) and left ventricular peak velocities of relaxation (negative dP/dt_max_).

***Determination of myocardial fatty acid, glycogen, and lactic acid***

After induction of CHF model in 4 weeks and training for 8 weeks, the rats were sacrificed and the heart was taken out and washed with normal saline. After drying with filter paper, the tissue was placed in liquid nitrogen and sorted at -80 ^°^C. The glycogen content, FA concentration, and the molar concentration of lactic acid in myocardial homogenate were determined by colorimetry according to the manufacturer’s instructions. 

***Quantitative real-time polymerase chain reaction***

Total RNA was extracted by using TRIzol reagent (Invitrogen; Thermo Fisher Scientific, Inc.) according to the manufacturer’s protocol, and cDNA was obtained by reverse transcription reaction with mRNA using an AccuPower RT PreMix kit (Bioneer Corporation, Daejeon, Korea). The mRNA levels of peroxisome proliferator activated receptor α (PPARα) and carnitine palmitoyltransferase 1 (CPT-1) were determined by quantitative real-time PCR (qRT-PCR). Primer sequences are shown in Table 1. Amplification conditions: pre-denaturation 95 ^°^C,1 min; 95 ^°^C, 30 sec; 55 ^°^C, 35 sec; 72 ^°^C, 10 min, totally 45 cycles. β-actin was used as an internal control to calculated relative expression. The relative RNA level was calculated using 2^△△^^Ct ^method.

***Western blot analysis***

Total protein was extracted from the experimental groups and the control group using RIPA lysis buffer (Beyotime Institute of Biotechnology, Jiangsu, China). A total of 60 μg of protein was separated by 10% SDS-PAGE and then transferred to the PVDF membrane. After blocking with 5% (w/v) milk in TBST [150 mM NaCl, 10 mM Tris-HCl (pH 7.5), and 0.1% Tween 20] at room temperature for 1 hr, the membrane was incubated with the primary antibody against collagen I (Abcam) [1:1,000 dilution], collagen III [1:1,000 dilution], transforming growth factor beta 1 (TGF-β1) [1:1,000 dilution], β-actin [1:2,000 dilution], AMPKα [1:1,000 dilution], phospho-AMPKα [1:1,000 dilution], glucose transporter type 4 (GLUT4) [1:1,000 dilution] or PPAR gamma coactivator (PGC) [1:1,000 dilution] at 4°C overnight. The PVDF membrane was washed with TBST, followed by incubation with secondary antibody (1:5000, MB005, Solarbio, Shanghai, China) for 1 hr at room temperature. Then a mixture of 2 ml substrate solution was added to PVDF membrane. After exposing, developing and fixing in darkroom, the protein band was visualized and analyzed for gray level analysis using ImageJ software (Bio-Rad, California, USA). The relative expression level of the target protein = the gray value of the target protein/β-actin protein.

***Histological examine and Masson’s Trichrome staining***

Tissue samples were embedded in paraffin and sectioned for staining. To evaluate the interstitial collagen deposition, sample slides were stained with Masson’s trichrome. For immunohistochemistry assay, samples were incubated with primary antibody against collagen I, collagen III or TGF-β1 for 12 hr at room temperature. After washing with phosphate-buffered saline, the slides were incubated with the corresponding secondary antibodies for 1 hr. The results were analyzed using the Image-Pro Plus 6.0 software. After the animals were euthanized, the hearts were harvested and stored at frozen in optimal cutting temperature (OCT) compound. Masson’s Trichrome staining was conducted to determine the infarct-scar size as described previously (8). 

***Statistical analysis***

All data were analyzed using SPSS16.0 statistical software (SPSS Inc. Chicago, IL, USA). The data were expressed as mean±standard deviation. Statistical analysis was performed by one-way ANOVA, and LSD test was used in paired comparison. A two-side *P*-value less than 0.05 was considered to be statistically significant.

## Results

***Infarcted area and cardiac remodeling***

By the end, the infarcted area size was around 36% in the CHF-Model group. Furthermore, the HW/BW ratio was higher in CHF-Model group compared to the Sham group (*P*<0.001), which is an indication of cardiac remodeling. Notably, the CHF-Model group also showed significant increases in both LV/BW and RV/BW ratios compared to the Sham group (*P*=0.007 and *P*=0.003, respectively, Table 2). 

**Table 1:** Primers for qRT-PCR

Gene	Length (bp)	Sequence
PPARα	510	P1 5’-AACACGGCATATCTTCGCTG-3’
		P2 5’-TCGGTCAGACTGAACCAGTG-3’
CPT-1	685	P1 5’-TATGATGTGAGGCTTTCGCC-3’
		P2 5’-CTCGGCTAAGAGGCTTAGTC-3’
β-actin	305	P1 5’-GAGACCACTTAACCCCCAG-3’
		P2 5’-GCCATCTTTCGCAATCGGTC-3’

**Table 2:** Changes in physiological parameters due to MI-induced CHF

	Sham group	CHF-Model group	P Values
Initial BW (g)	208±6	217±12	0.36
Final BW (g)	316±9	308±8	0.27
HR (bpm)	287±25	264±19	0.62
MAP (mmHg)	128±6	113±8	0.02
HW/BW (mg⁄g)	3.21±0.09	4.03±0.16	0.001
LV/BW (mg⁄g)	2.77±0.09	3.26±0.15	0.007
RV/BW (mg⁄g)	0.61±0.04	1.12±0.07	0.003
LW/BW (mg⁄g)	1.80±0.03	2.53±0.07	0.001

BW: body weight; HR: heart rate; MAP: mean arterial pressure; LV: left ventricle; RV: right ventricle

**Table 3:** Changes in physiological parameters due to MI-induced CHF

Group	n	LVSP/mmHg	LVEDP/mmHg	Positive dP/dt_max_ /(mmHg/s)	negative dP/dt_max_ /(mmHg/s)
Sham group	20	134.41±10.78	4.32±1.54	4317.55±443.20	4227.16±377.54
CHF-Model group	20	77.43±9.63#	12.06±2.51#	2453.49±203.67#	2027.56±191.03#
Exercise-CHF group	20	109.01±12.45*#	7.61±1.52*#	3665.74±290.31*#	2824.32±252.47*#

* *P<*0.05, compared with CHF-Model group;

# *P<*0.05, compared with Sham group

**Table 4:** Effects of AIT intervention on glycogen

Group	n	Glycogen/(mg/g)	FA/(mmol/L)	Lactic acid/(μmmol/g)
Sham group	20	4.21±1.42	0.64±0.07	125.21±25.44
CHF-Model group	20	2.42±0.78#	1.81±0.30#	304.21±48.65#
Exercise-CHF group	20	3.74±0.65*	0.76±0.24*	229.14±33.05*#

* *P<*0.05, compared with CHF-Model group;

# *P<*0.05, compared with Sham group

**Figure 1:**
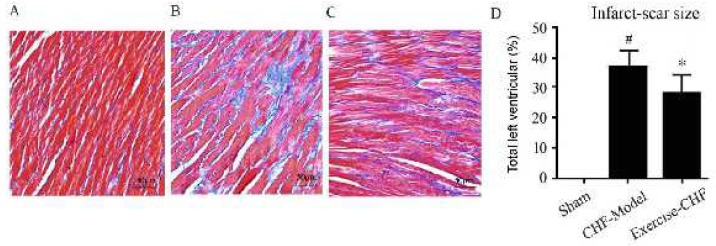
Effects of aerobic interval training (AIT) on fibrosis in the myocardium of myocardial infarction (MI)-induced chronic heart failure (CHF) rats by Masson’s trichrome. A: Sham group; B: CHF-Model group; C: Exercise-CHF group; D: Quantitative analysis of infarct-scar size. #*P<*0.05 vs sham group, *P<0.05 vs CHF-Model group

**Figure 2:**
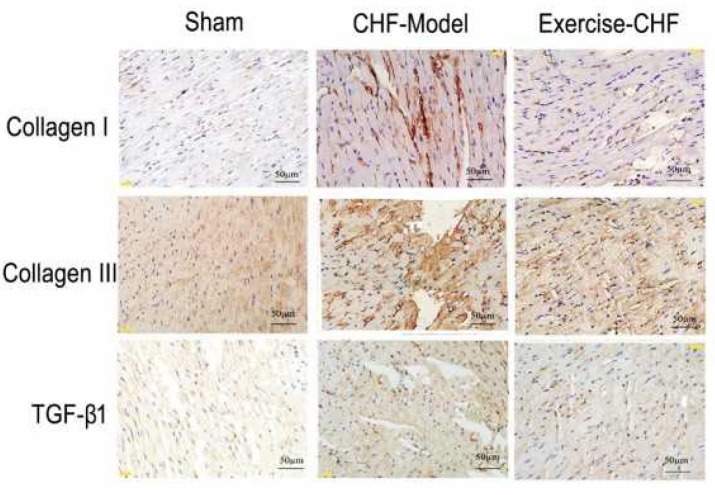
Effects of aerobic interval training (AIT) on collagen deposition in the myocardium of myocardial infarction (MI)-induced chronic heart failure (CHF) rats. Expression levels of collagen I, collagen III and transforming growth factor beta 1 (TGF-β1) in the Sham, CHF-model and Exercise-CHF groups were determined by immunohistochemical staining

**Figure 3:**
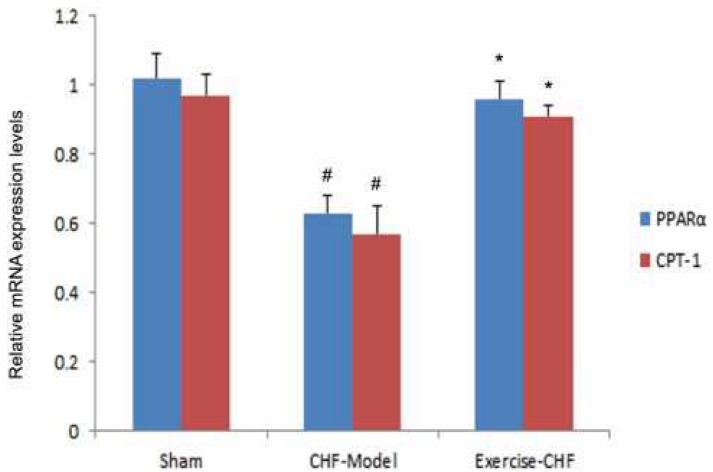
Effects of aerobic interval training (AIT) on mRNA expression levels of peroxisome proliferator activated receptor α (PPARα) and carnitine palmitoyltransferase 1 (CPT-1) in myocardium of myocardial infarction (MI)-induced chronic heart failure (CHF) rats. mRNA levels of PPARα and CPT-1 in different groups were determined by qRT-PCR. #*P<*0.05 vs sham group, *P<0.05 vs CHF-Model group

**Figure 4:**
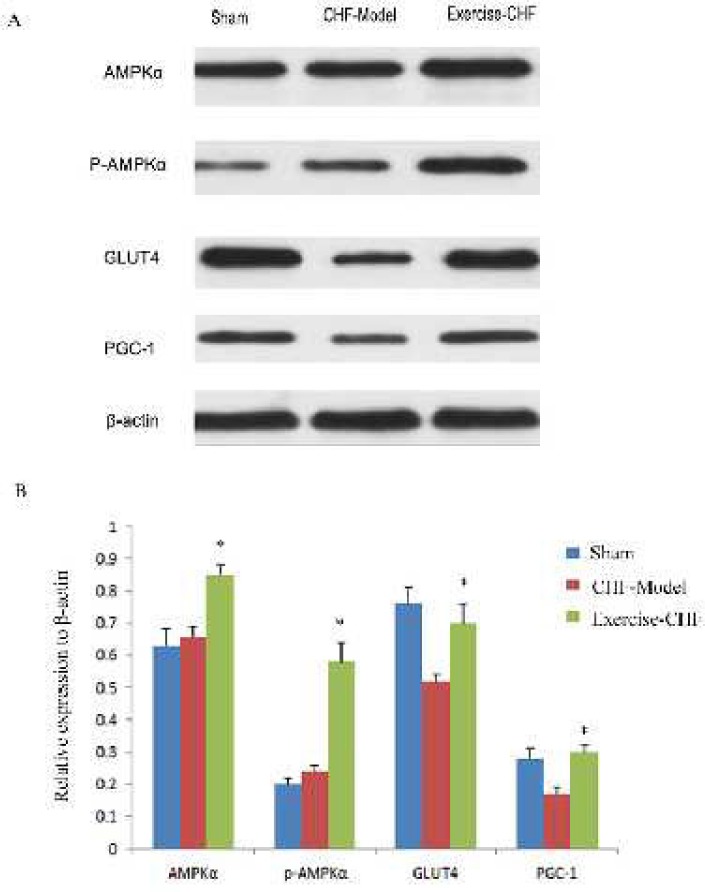
Effects of aerobic interval training (AIT) on expression and phosphorylation of key components in AMP-activated protein kinase/peroxisome proliferator activated receptor α (AMPK/PPARα) signaling pathway in myocardium of myocardial infarction (MI)-induced chronic heart failure (CHF) rats. Levels of AMPKα, pAMPKα, glucose transporter type 4 (GLUT4) and PPAR gamma coactivator (PGC) in different group were determined by western blot with the indicated antibodies

***Effects of aerobic interval training on cardiac function in chronic heart failure rats***

Compared to the Sham group, the LVSP, maximal positive and negative dP/dt, were significantly reduced (*P*=0.036), while LVEDP was significantly increased (*P*<0.05) in the CHF-Model group. In the Exercise-CHF group, the LVSP, maximal positive and negative dP/dt, were significantly increased (*P*<0.05), and LVEDP was significantly reduced (*P*<0.05, Table 3) compared to the CHF-Model group. 

***Changes of myocardial glycogen, fatty acid, and lactic acid***

Compared to the Sham group, the glycogen content was significantly decreased (*P*<0.05), while the FA concentration and the content of lactic acid were significantly increased in the CHF-Model group (*P*<0.05). In the Exercise-CHF group, the glycogen content was significantly increased (*P*<0.05), and the FA concentration and the content of lactic acid were significantly decreased (*P*<0.05) compared to the CHF-Model group (Table 4).

***Pathological changes of heart tissues***

Masson’s trichrome staining showed increased fibrosis in the interstitial regions of the CHF-Model group compared to the Sham group. AIT significantly reduced the infarct size compared to the CHF-Model group (26.57±2.73% vs 38.03±2.42%) (Figure 1, *P*<0.05). Immunohistochemical staining results showed that AIT significantly decreased the collagen deposition in heart tissues, as demonstrated by decreased expression levels of collagen I, collagen III, and TGF-β1 (Figure 2). 

***Changes of myocardial PPARα and CPT-1 mRNA***

qRT-PCR results showed that the mRNA expression levels of PPARα and CPT-1 in the CHF-Model group were significantly lower than those of the Sham group (*P*<0.05). But, AIT could restore the mRNA levels of PPARα and CPT-1 (*P*<0.05, Figure 3). 

***Changes of protein and phosphorylation levels of AMPKα, GLUT4, and PGC-1α in myocardium***

There was no significant difference of total AMPKα protein among the groups (*P*>0.05). AMPKα phosphorylation level was highest in the Exercise-CHF group, followed by the CHF-Model group, and was lowest in the Sham group (*P*<0.05, Figure 4). The change trends of GLUT4 and PGC-1 levels were consistent. GLUT4 and PGC-1 protein levels were lower in the CHF-Model group when compared to the Sham group (*P*<0.05), while AIT restored the expression levels of GLUT4 and PGC-1 (Figure 4). 

## Discussion

In this study, we successfully established the CHF model, in which a typical post-infarction ventricular diastolic function decreased. Previous studies have reported that exercise training before acute MI reduces the infarct scar size and improves heart function in rats. Interestingly, in this study, we found that AIT after acute MI decreased infarct scar size as well. Infarct scar is a requisite to the rebuilding of necrotic myocardium following MI (9). Post-infarction healing has been considered to complete 6–8 weeks following MI (10, 11) that results in scar tissue containing fibrillar collagen and myofibroblasts. With the CHF model, we showed that exercise could promote FA utilization, glucose uptake, and aerobic oxidation, increase myocardial mitochondrial biogenesis, improve myocardial aerobic capacity, and reduce heart lipid deposition and lactic acid accumulation, thus leading to improved metabolic cardiac remodeling and cardiac function after CHF. A sham group was used as a control, and the sham-Exercise was excluded in this study as we observed no significant difference between these two groups. After 8 weeks of AIT, the cardiac function of the Exercise-CHF group was improved, as demonstrated by significantly decreased LVEDP and FA and lactic acid content, and increased LVSP, maximal positive and negative dP/dt, and elevated glycogen content. These results suggest that during post-MI CHF cardiac metabolic remodeling, energy production is reduced, and acidosis and lipid toxicity cause further reduction of heart function. Aerobic exercise can delay the pathological process, which is consistent with the researches from clinical epidemiology (12, 13). 

AMPK is involved in the development of heart and myocardial metabolism, and plays a protective role in myocardial ischemia reperfusion injury (14). In this study, after 8 weeks of AIT, the total AMPKα protein level did not change, but phosphorylation of AMPKα in the Exercise-CHF group was significantly higher than that of the CHF-Model group or Sham group, indicating that AIT sustained up-regulated AMPKα activity. This finding is similar to the results that long-term exercise improves AMPKα activity in skeletal muscle, fat, and liver (15, 16).

PGC-1α is a key signal molecule regulating mitochondrial biogenesis. Its increased expression activates NRFs and Tfam transcription factor, and mitochondrial DNA replication and transcription, which induce mitochondrial biogenesis (17). In contrast, the levels of p-AMPKα and PGC-1α in the Exercise-CHF group were significantly increased, suggesting that AIT could activate AMPK-PGC-1 pathway and promote the biosynthesis of myocardial mitochondria (18).

Up-regulation of PGC-1α has been reported to increase mitochondrial volume density and be positively correlated with the changes of VO_2_max and anaerobic threshold in patients with CHF (19). Therefore, exercise-induced up-regulation of AMPK-PGC-1α improves energy production during CHF, and also increases systolic function and aerobic capacity (VO_2_max) (20), which lay the material foundation for the benign changes of metabolic remodeling. Findings have shown that PPARα is a downstream target molecule of AMPK, and AMPK can up-regulate the expression of PPARα. They form AMPK-PPARα signaling pathway, which plays an important role in maintaining the stability of the myocardial energy metabolism (21, 22). In this study, we found that although the AMPK was increased, the PPARα mRNA was significantly lower in the CHF-Model group compared to the Sham group, indicating that a slight activation of AMPK is not enough to up-regulate the PPARα or other network signaling pathways, such as PGC1α.

Along with the decreased CPT-1 expression, myocardial FA content increased significantly, which resulted in reduced FA utilization in CHF. In CHF, FA supply is reduced, which reduces myocardial oxygen consumption under ischemia hypoxia, but at the same time it also results in lipid deposition in cardiac, which induces myocardial apoptosis and cardiac abnormalities by lipotoxicity.

In this study, GLUT4 protein of the Exercise-CHF group was significantly higher than that of the CHF-Model group, suggesting that AIT activates AMPK and elevates GLUT4 expression, and thus increases glucose uptake. Meanwhile, the myocardial lactate content of the Exercise-CHF group was significantly lower than that of the CHF-Model group, but still higher than that of the Sham group, which suggested that AIT could promote the transformation of glycolysis to aerobic oxidation in CHF. The possible mechanism is that the long-term movement increase blood flow and aerobic metabolism ability through increased mitochondrial biogenesis, and diastolic blood vessels. We demonstrated that AIT improves myocardial energy metabolism in CHF rats, along with the corresponding alteration in metabolism-related genes. Our findings might provide new insights into the protective role of physically activity against CHF. 

## Conclusions

Taken together, AIT improves the metabolic remodeling of cardiac CHF and restores cardiac function, which may be associated with activation of AMPK and its downstream signaling pathway. 
